# A cost-effectiveness study of PSMA-PET/CT for the detection of clinically significant prostate cancer

**DOI:** 10.1007/s00259-025-07190-6

**Published:** 2025-03-12

**Authors:** Bastiaan M. Privé, Tim M. Govers, Bas Israël, Marcel J. R. Janssen, Bart J. R. Timmermans, Steffie M. B. Peters, Michel de Groot, Patrik Zámecnik, Stan R. W. Wijn, Alexander Hoepping, J. P. Michiel Sedelaar, Jelle O. Barentsz, Inge M. van Oort, Maarten de Rooij, James Nagarajah

**Affiliations:** 1 Department of Medical Imaging, Radboud University Medical Center, Radboud Institute for Health Sciences, Nijmegen, The Netherlands; 2 Department of Radiation Oncology, Erasmus Medical Center, Cancer Institute, Rotterdam, The Netherlands; 3 Medip Analytics, Nijmegen, The Netherlands; 4 Department of Urology, Radboud University Medical Center, Radboud Institute for Health Sciences, Nijmegen, The Netherlands; 5 Department of Radiation Oncology, Radboud University Medical Center, Radboud Institute for Health Sciences, Nijmegen, The Netherlands; 6 Department of Medicinal Chemistry, ABX Advanced Biochemical Compounds Gmbh, 1454 Radeberg, Germany; 7 Department of Medical Imaging Andros Clinics, Arnhem, The Netherlands

**Keywords:** Cost-effectiveness, Prostate cancer, PSA, PSMA, mpMRI

## Abstract

**Background:**

Prostate-specific membrane antigen (PSMA) positron emission tomography/computed tomography (PET/CT) is currently under evaluation for detecting clinically significant prostate cancer. The PSMA-PET/CT may complement the current standard diagnostic pathway for prostate cancer, which includes prostate-specific antigen (PSA) testing and multiparametric magnetic resonance imaging (mpMRI). This study evaluated the cost-effectiveness and quality of life impact of incorporating PSMA-PET/CT into this diagnostic algorithm.

**Methods:**

A life-time decision model compared the current standard of care of a MRI driven diagnostic pathway, where men undergo prostate biopsy in case of a Prostate Imaging Reporting and Data System (PI-RADS) scores 3–5, to a strategy incorporating PSMA-PET/CT to potentially avoid unnecessary biopsies. Long-term quality-adjusted life years (QALY) and healthcare costs were calculated for each approach.

**Results:**

In PI-RADS 3 lesions, PSMA-PET/CT improved the per-patient QALY by 0.002 and was borderline cost-effective, with an increased cost of €170-€186 per patient and an incremental cost-effectiveness ratio (ICER) of €56,700-€93,212 per QALY. In PI-RADS 1–2, additional biopsies and over-detection of low-risk prostate cancers led to a per-patient QALY decrease of 0.001 points, a cost increase of €416-€429 per patient and was thus not cost-effective.

**Conclusion:**

The addition of PSMA-PET/CT to MRI in patients with equivocal MRI findings appears to be borderline cost-effective due to biopsy avoidance and a reduced detection of indolent, low-risk tumors. In men with a negative MRI, adding a PSMA-PET/CT does not seem to be cost-effective due to a higher number of unnecessary biopsies and only minor improvement in the detection of clinically significant prostate cancer.

**Supplementary Information:**

The online version contains supplementary material available at 10.1007/s00259-025-07190-6.

## Introduction

Prostate cancer is the most common non-skin malignancy in males globally, with an incidence of 1.4 million new cases and 375.000 deaths annually [1]. The disease poses a significant economic burden, with yearly macroeconomic costs estimated of approximately 564 billion euros [2]. The aging population and advances in healthcare are likely to increase healthcare costs in the near future. To maintain sustainable healthcare systems, new interventions must demonstrate cost-effectiveness.

Prostate cancer significantly impacts patient lifespan and quality of life, prompting many aging males to undergo regular prostate-specific antigen (PSA) testing to potentially improve outcomes [3, 4]. However, elevated PSA levels may lead to prostate biopsies, which can result in adverse effects such as pain, infection, and lower urinary tract symptoms [5]. Annually, over 3 million patients undergo these biopsies [6]. The integration of prostate magnetic resonance imaging (MRI) has significantly improved the diagnostic pathway of prostate cancer. Prostate MRI effectively reduces unnecessary biopsies and enables a more targeted approach when biopsy is indicated. Consequently, the PSA-MRI diagnostic pathway has demonstrated cost-effectiveness compared to PSA testing alone [7, 8]. However, the MRI pathway still has limitations. Equivocal MRI outcomes, classified as Prostate Imaging Reporting and Data System (PI-RADS) 3, present diagnostic challenges, and false-negative results in PI-RADS 1–2 cases can occur, despite the modality's high overall sensitivity. These limitations underscore the need for further refinement of the MRI diagnostic algorithm to optimize its clinical utility and cost-effectiveness in prostate cancer detection and management.

Prostate-specific membrane antigen (PSMA) positron emission tomography/computed tomography (PET/CT) has emerged as the standard of care for evaluating locoregional and metastatic prostate cancer [9, 10]. Presently, there is also incremental interest of PSMA-PET/CT to evaluate the local prostate for cancer. Two prospective trials reported improved outcomes using a MRI + PSMA-PET/CT diagnostic pathway, detecting additional cancers in PI-RADS 1–2 cases and potentially reducing unnecessary biopsies in PI-RADS 3 lesions [11, 12]. As a result, a non-inferiority trial called PRIMARY2 (NCT05154162) is currently recruiting patients (13). In this study, patients with high risk PI-RADS 2 and PI-RADS 3 index lesions, will undergo biopsy in case of positive findings on an additional PSMA-PET/CT. Yet, it is still unknown whether this diagnostic strategy is cost-effective and if it improves the quality of life.

Therefore, the aim of this study was to investigate the cost-effectiveness and quality of life benefit of adding PSMA-PET/CT into the MRI driven diagnostic pathway for prostate cancer.

## Materials and methods

This is a sub-analysis of a study that was approved by the Medical Review Ethics Committee Arnhem-Nijmegen (NL73559.091.20) and was registered on clinicaltrials.gov (NCT04487847). All participants provided written informed consent, and the study was conducted in accordance with Good Clinical Practice and Declaration of Helsinki guidelines.

The target population for this study comprises of biopsy naïve men with elevated PSA levels and/or abnormal digital rectal exams who underwent prostate MRI as part of standard clinical care. The diagnostic accuracy of the prostate MRI were derived from the initial paper [12] and for external validation, from Drost et al. [13]. Data to assess the additional value of the PSMA-PET/CT were extracted from the initial study (12) and for external validation, from a previously reported prospective study called PRIMARY1 (11). Each cancer was grouped by low-, intermediate- and high-risk as defined by D’Amico [14]. Diagnostic performance of prostate MRI and PSMA-PET/CT scan are provided in Tables 1, 2, 3 and 4. The standard of clinical care (SoC) involves systematic and MR target biopsy in patients with PI-RADS scores ≥ 3. In the tested strategy, those with level of suspicion (LOS) ≥ 3 would undergo biopsy. No biopsy was performed in PI-RADS 1–2 and LOS 1–2.

**Table 1:** Internal validation: MRI results from the initial study [12]

Disease classification	PI-RADS 1–2	PI-RADS 3	PI-RADS 4–5
*No prostate cancer (specificity), %*	51	30	19
*Low-risk prostate cancer (sensitivity), %*	9	18	73
*Intermediate-risk prostate cancer (sensitivity), %*	11	0	89
*High-risk prostate cancer (sensitivity), %*	0	33	67

**Table 2:** External validation: MRI results from literature [13]

*Disease classification*	PI-RADS 1–2	PI-RADS 3	PI-RADS 4–5
*No prostate cancer (specificity), %*	38	34	28
*Low-risk prostate cancer (sensitivity), %*	30	26	44
*Intermediate-risk prostate cancer (sensitivity), %*	14	14	72
*High-risk prostate cancer(sensitivity), %*	4	4	92

**Table 3:** Internal validation: PSMA-PET/CT results from the initial study [12]

Disease classification	LOS 1–2	LOS 3	LOS 4–5
*No prostate cancer (specificity), %*	70	14	16
*Low-risk prostate cancer (sensitivity), %*	27	9	64
*Intermediate-risk prostate cancer (sensitivity), %*	11	0	89
*High-risk prostate cancer (sensitivity), %*	0	0	100

**Table 4:** External validation: PSMA-PET/CT results from literature [11]

Disease classification	Data from paper
*No prostate cancer (specificity), %*	50
*Low-risk prostate cancer (specificity), %*	50
*Intermediate-risk prostate cancer (sensitivity), %*	90
*High-risk prostate cancer (sensitivity), %*	90

*CT*, computed Tomography; *ICER*, Incremental cost-effectiveness ratio; *LOS*, Level of suspicion; *PET*, Positron Emission Tomography; *PI-RADS*, Prostate Imaging Reporting and Data System; *PSMA*, Prostate-Specific Membrane Antigen

### Model

The analyses were performed using the Medip Prostate Dx Application version 1.1.0, Medip Analytics, Nijmegen, The Netherlands. The Medip Prostate Dx Application is a dynamic software application which offers a solution for interaction with an underlying dynamic individual-level patient simulation model of the prostate cancer pathway. Within the software the comparator (i.e. standard of care) can be customized as well as the position and characteristics of the strategy under investigation.

### Technical model details

The model underlying the Medip Prostate Dx Application is a combination between a decision tree and state transition model. Individual patients are simulated with a disease status and age. The decision tree is used to resemble the chosen diagnostic and initial treatment pathway. The health state transition model resembles the follow-up of the patients and assesses the consequences of the outcomes of the diagnostic and initial treatment pathway. It is a life-time model to predict outcome of individual patients until they die.

**Fig. 1:**
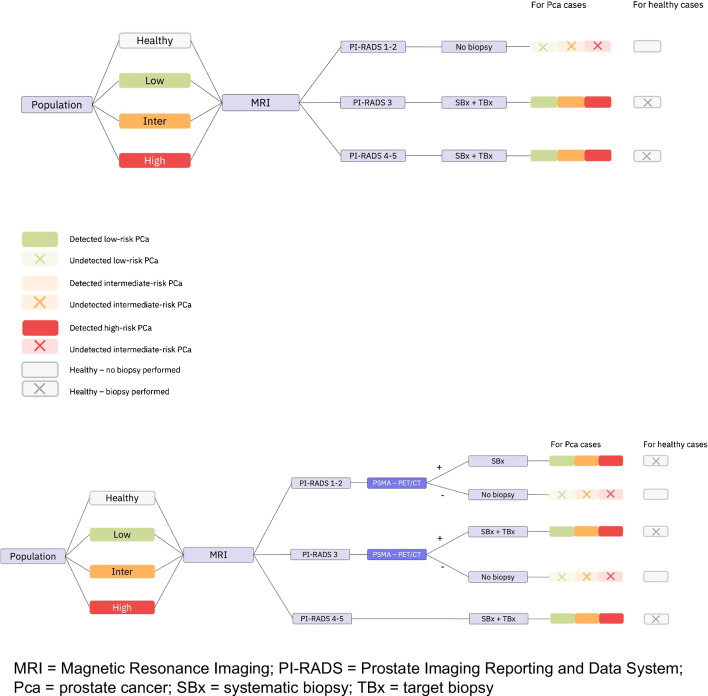
Decision tree for SOC and strategy with PSMA-PET/CT. MRI = Magnetic Resonance Imaging; PI-RADS = Prostate Imaging Reporting and Data System; Pca = prostate cancer; SBx = systematic biopsy; TBx = target biopsy

**Fig. 2:**
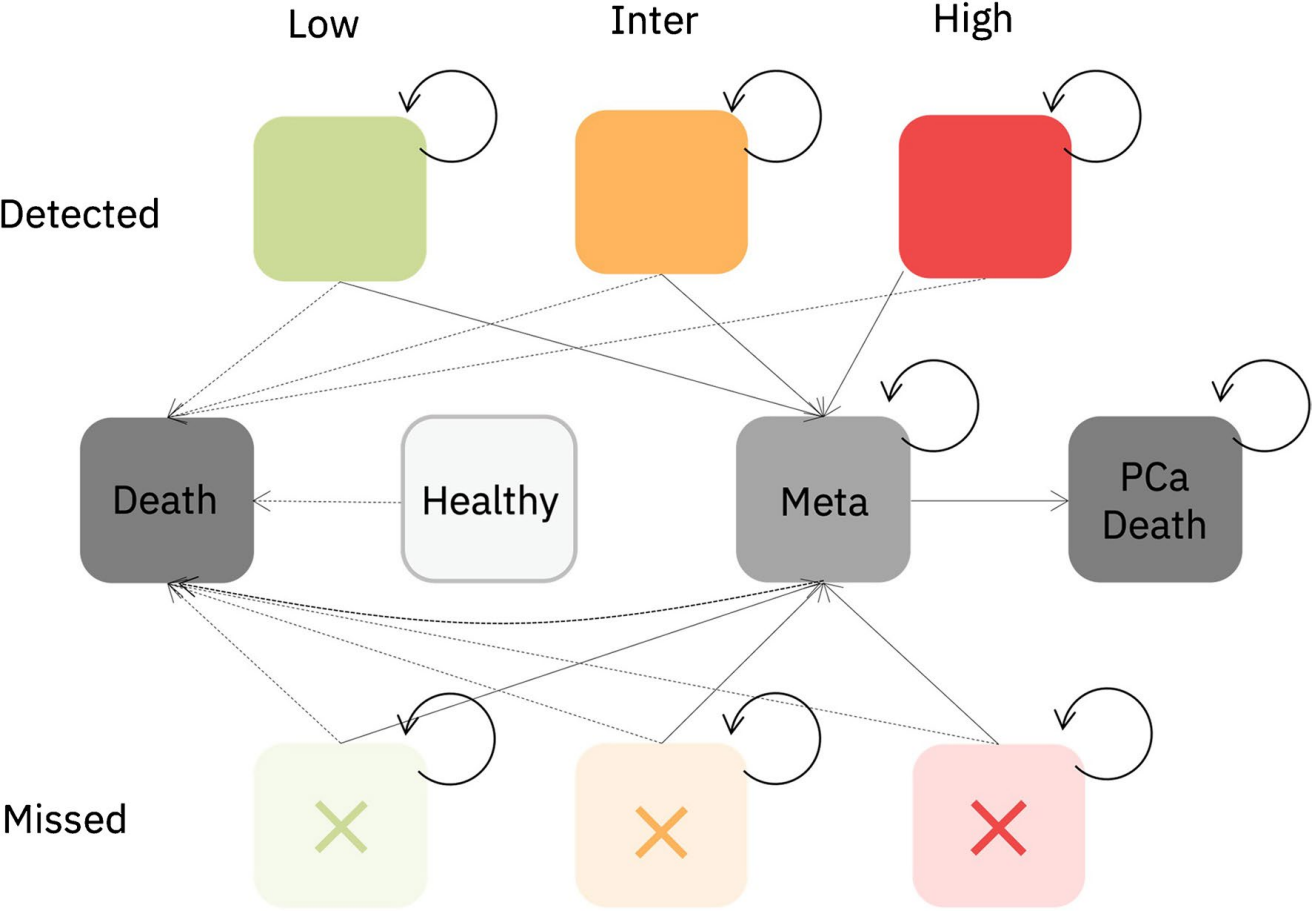
Health state transition model

The model simulates patients with no prostate cancer, as well as those with low-, intermediate-, or high-risk prostate cancer. Based on the accuracy of the chosen diagnostic strategy, healthy patients can be classified as true negative (correctly identified) or false positives (those who undergo unnecessary biopsy). Patients with prostate cancer can be either detected or missed. The model assigns treatments to both detected and missed patients based on a predefined distribution of treatments. Patients may progress to metastatic disease depending on their detection and treatment status, and those with metastatic disease patients may die from prostate cancer. Quality of life after diagnosis and treatment is assessed based on disutility’s associated with interventions, potential side effects and the status of disease progression. Costs are associated with diagnostic modalities, treatments, side effects and disease progression. A schematic overview of the model is presented in Fig. 1, which illustrated the decision tree, and Fig. 2, which depicts the state transition model.

### Inputs

Model inputs include disease prevalence, treatment distributions, side effects, costs, quality of life values, progression rates and mortality. These inputs are listed in Table 5. Costs of the PSMA-PET/CT were estimated at €1,129. As is standard in cost-effectiveness studies, costs were discounted with 4% and effects by 1.5%. The supplementary material provide tornado plots showing the effect on study outcomes using a 25% difference of the original input.

**Table 5:** Model inputs

Parameter	Value	Resource
*Prevalence of prostate cancer*	49%	Drost et al. 2019 [13]
-Of which low-risk	41%	Drost et al. 2019 [13]
-Of which intermediate-risk	30%	Drost et al. 2019 [13]
-Of which high-risk	29%	Drost et al. 2019 [13]
*Treatments low-risk*		
Active surveillance	73%	Evers et al. 2022 [18]
RARP	15%	Evers et al. 2022 [18]
IMRT	6%	Evers et al. 2022 [18]
Brachytherapy	6%	Evers et al. 2022 [18]
*Treatments intermediate-risk*		
Active surveillance	25%	Evers et al. 2022 [18]
RARP	33%	Evers et al. 2022 [18]
IMRT	32%	Evers et al. 2022 [18]
Brachytherapy	10%	Evers et al. 2022 [18]
*Treatments high-risk*		
RARP	33%	Evers et al. 2022 [18]
IMRT	45%	Evers et al. 2022 [18]
Hormone therapy	7%	Evers et al. 2022 [18]
Watchful waiting	15%	Evers et al. 2022 [18]
*Side effects*		
Biopsy – Infection (with hospitalization)	1%	Lundstrom et al. 2014 [19]
RARP—incontinence	9%	Cooperberg et al. 2013 [20]
RARP—Erectile dysfunction	28%	Cooperberg et al. 2013 [20]
IMRT – Erectile dysfunction	26%	Cooperberg et al. 2013 [20]
IMRT – GI Toxicity	6%	Viani et al. 2016 [21]
IMRT – GU Toxicity	4%	Viani et al. 2016 [21]
Brachy – Erectile disfunction	17%	Cooperberg et al. 2012 [20]
Brachy – GI Toxicity	4%	Cooperberg et al. 2012 [20]
Brachy – GU Toxicity	1%	Cooperberg et al. 2012 [20]
Hormonal therapy – Erectile dysfunction	69%	Gryzinski et al. 2022 [22]
*Quality of life (disutility’s)*		
* Infection after biopsy (once)*	−0.28	Keeney et al. 2022 [23]
* Biopsy general (once)*	−0.006	Keeney et al. 2022 [23]
* Erectile dysfunction (per year)*	−0.1	Stewart et al. 2005 [24]
* Incontinence (per year)*	−0.16	Stewart et al. 2005 [24]
* GI Toxicity (per year)*	−0.28	Stewart et al. 2005 [24]
* GU Toxicity (per year)*	−0.1	Stewart et al. 2005 [24]
* Active surveillance & watchful waiting (per year)*	−0.03	Heijnsdijk et al. 2012 [25]
* Metastases (per year)*	−0.4	Heijnsdijk et al. 2012 [25]
*Costs*		
Urology visit	€126	Venderink et al. 2017 [26]
MRI	€400	Venderink et al. 2017 [26]
Biopsy	€606	Venderink et al. 2017 [26]
Infection with hospitalization	€3,165	Venderink et al. 2017 [26]
RARP	€11,866	Venderink et al. 2017 [26]
IMRT	€10,947	Venderink et al. 2017 [26]
Brachytherapy	€11,865	Venderink et al. 2017 [26]
Hormone therapy	€2,748	Venderink et al. 2017 [26]
Active surveillance (year 1)	€572	Venderink et al. 2017 [26]
Active surveillance (year 2 +)	€306	Venderink et al. 2017 [26]
Watchful waiting (per year)	€126	Venderink et al. 2017 [26]
Incontinence year 1	€804	Venderink et al. 2017 [26]
Incontinence year 2 +	€33	Venderink et al. 2017 [26]
GI toxicity (once)	€2,545	Venderink et al. 2017 [26]
GU toxicity (once)	€2,365	Venderink et al. 2017 [26]
Metastases (per year)	€24,074	Calculation (Supplement A)
Death due to prostate cancer	€30,071	Kok et al. 2009
Death due to other causes	€22,412	Kok et al. 2009
*Progression to metastases*		
Low-risk detected—5 year	2%	Wilt et al. 2017 [27]
Low-risk detected—10 years	3%	Wilt et al. 2017 [27]
Low-risk detected—15 years	6%	Wilt et al. 2017 [27]
Intermediate-risk detected – 5 years	2%	Wilt et al. 2017 [27]
Intermediate-risk detected – 10 years	4%	Wilt et al. 2017 [27]
Intermediate-risk detected – 15 years	9%	Wilt et al. 2017 [27]
High-risk detected – 5 years	1%	Wilt et al. 2017 [27]
High-risk detected – 10 years	4%	Wilt et al. 2017 [27]
High-risk detected – 15 years	14%	Wilt et al. 2017 [27]
Low-risk undetected—HR	1.85	Wilt et al. 2017 [27]
Intermediate-risk undetected—HR	2.38	Wilt et al. 2017 [27]
High-risk undetected—HR	2.70	Wilt et al. 2017 [27]

*GI*, gastro-intestinal; *GU*, genito-urinary; *IMRT*, intensity modulated radiotherapy; *MRI*, magnetic resonance imaging; *RARP*, robot assisted radical prostatectomy

### Study outcomess

The primary objective was to evaluate the cost-effectiveness and outcome on quality of life of acquiring an additional PSMA-PET/CT following prostate MRI in the case of a PI-RADS 3 index lesion. For the primary objective two models were used: the original diagnostic accuracy of the MRI from the initial study (internal validation), or the MRI data as described by literature, i.e. by Drost et al. (external validation) [13]. Secondary objectives were: the cost-effectiveness and outcome on quality of life of acquiring a PSMA-PET/CT following MRI in the setting of a negative MRI (PI-RADS 1–2) and PI-RADS 1–3. To externally validate the outcomes of our trial data, we also modeled cost-effectiveness and outcome on quality of life using data from an external PSMA-PET/CT study (PRIMARY1 trial) [11]. Quality-adjusted life years (QALYs) were used as the effectiveness outcome. When applicable incremental cost-effectiveness ratios (ICER) were calculated. A willingness-to-pay of 80,000 euros per QALY was used to define whether a strategy was deemed cost-effective against its comparator.

## Results

The internal and external study included 75 and 291 patients, respectively [11, 12]. At inclusion, the median PSA was 7 (interquartile range [IQR] 4.9 – 10) and 5.6 (IQR 4.2 – 7.5), respectively. The supplementary material provides a table of more baseline demographics of both trials. For detailed descriptions of the study cohorts and outcomes, we refer to the original manuscripts [11, 12]. In short, all patients received mpMRI and PSMA-PETCT/CT prior to biopsy. With the gold-standard of histopathology, the sensitivity, specificity, positive predictive value and negative predictive value were calculated. Tables 1, 2, 3 and 4 presents the diagnostic accuracy of the MRI and PSMA-PET/CT according to the D’Amico scoring system [14].

In the setting of a PI-RADS 3 index lesion, PSMA-PET/CT could have prevented biopsies in 107 or 121 patients (out of 1000 patients), following the initial study (internal validation of MRI) and literature on MRI (external validation of MRI) [13], respectively (Table 6). In this setting, 10 or 14 (out of 1000 patients) cases of low-risk prostate cancer would have remained undetected, respectively, and potentially two intermediate-risk prostate cancers (out of 1000 patients). With the present data, no high-risk prostate cancers would have been missed. With this strategy, the overall per patient QALY improved by 0.002. Between 0 and 2 extra patients (out of 1000) would have developed metastases. The strategy is borderline cost-effective with an increased cost of €170-€186 per patient resulting in an ICER of €56,700 to €93,212 per QALY.

**Table 6:** Clinical effects of the MRI and PSMA-PET/CT diagnostic pathway per 1000 patients

Internal MRI and PSMA-PET/CT data [12]	PI-RADS 1–2	PI-RADS 3	PI-RADS 1–3
* Additional biopsies, n (%)*	+ 78 (15)	- 107 (21)	- 29 (6)
* Low-risk prostate cancer, n (%)*	+ 13 (6)	- 10 (5)	+ 3 (1)
* Intermediate-risk prostate cancer, n (%)*	+ 14 (10)	0 (0)	+ 14 (10)
* High-risk prostate cancer, n (%)*	+ 0 (0)	0 (0)	0 (0)
External MRI data [13] + internal PSMA-PET/CT data [12]	**PI-RADS 1–2**	**PI-RADS 3**	**PI-RADS 1–3**
* Additional biopsies, n (%)*	+ 58 (11)	- 121 (24)	- 63 (12)
* Low-risk prostate cancer, n (%)*	+ 44 (22)	- 14 (7)	+ 30 (15)
* Intermediate-risk prostate cancer, n (%)*	+ 19 (13)	- 2 (1)	+ 16 (11)
* High-risk prostate cancer, n (%)*	+ 6 (4)	0 (0)	+ 6 (4)
External MRI and PSMA-PET/CT data [11, 13]	**PI-RADS 1–2**	**PI-RADS 3**	**PI-RADS 1–3**
* Additional biopsies, n (%)*	+ 97 (19)	- 87 (17)	+ 10 (2)
* Low-risk prostate cancer, n (%)*	+ 30 (15)	- 26 (13)	+ 4 (2)
* Intermediate-risk prostate cancer, n (%)*	+ 19 (13)	- 2 (1)	+ 17 (12)
* High-risk prostate cancer, n (%)*	+ 5 (4)	0 (0)	5 (4)

*CT*, computed Tomography; *PET*, Positron Emission Tomography; *PI-RADS*, Prostate Imaging Reporting and Data System; *PSMA*, Prostate-Specific Membrane Antigen

When an additional PSMA-PET/CT is performed in PI-RADS 1–2, 58 or 78 (out of 1000 patients) additional biopsies would have been performed following internal validation and external validation of MRI, respectively. This would result, in the diagnosis of 13 or 44 (out of 1000 patients) additional low-risk prostate cancers, an additional 14 or 19 (out of 1000 patients) intermediate-risk prostate cancers and 0 or 6 high-risk prostate cancers (out of 1000 patients). This would have prevented the development of metastases in 3 or 6 patients in 1000 patients. Yet, due to additional biopsies and additional detection of low-risk prostate cancers, the per patient QALY drops by 0.001 points. As the costs would increase with €416-€429 per patient, the strategy is deemed not cost-effective. Cumulative numbers for PI-RADS 1–2 and PI-RADS 3 can be seen in Tables 7, 8 and 9. This table also includes the outcomes using the PRIMARY1 data as an input.

**Table 7:** Quality of life benefit and cost-effectiveness using internal MRI and PSMA-PET/CT data [12]

	Standard of care	PSMA-PET/CT strategy	Difference
PI-RADS 1–2			
* Quality adjusted life years*	12.974	12.973	−0.001
* Metastases, n*	77	74	−3
* Diagnostic costs, €*	€ 976,00	€ 1.376,00	€ 400,00
* Treatment costs, €*	€ 3.130,00	€ 3.308,00	€ 178,00
* Long term costs, €*	€ 18.564,00	€ 18.402,00	-€ 162,00
* Total disease costs, €*	€ 22.670,00	€ 23.086,00	€ 416,00
* ICER*	0	Not applicable	
PI-RADS 3			
* Quality adjusted life years*	12.974	12.976	+ 0.002
* Metastases, n*	77	77	0
* Diagnostic costs, €*	€ 976,00	€ 1.167,00	€ 191,00
* Treatment costs, €*	€ 3.130,00	€ 3.095,00	-€ 35,00
* Long term costs, €*	€ 18.564,00	€ 18.578,00	€ 14,00
* Total disease costs, €*	€ 22.670,00	€ 22.840,00	€ 170,00
* ICER*	0	€ 56.700,00	Cost-effective
PI-RADS 1–3			
* Quality adjusted life years*	12.974	12.976	+ 0.002
* Metastases, n*	77	74	−3
* Diagnostic costs, €*	€ 976,00	€ 1.567,00	€ 591,00
* Treatment costs, €*	€ 3.130,00	€ 3.273,00	€ 143,00
* Long term costs, €*	€ 18.564,00	€ 18.415,00	-€ 149,00
* Total disease costs, €*	€ 22.670,00	€ 23.256,00	€ 586,00
* ICER*	0	€ 293.095,00	Not cost-effective

*CT*, computed Tomography; *ICER*, Incremental cost-effectiveness ratio; *MRI*, magnetic resonance imaging; *PET*, Positron Emission Tomography; *PI-RADS*, Prostate Imaging Reporting and Data System; *PSMA*, Prostate-Specific Membrane Antigen

**Table 8:** Quality of life benefit and cost-effectiveness using external MRI [13] and internal PSMA-PET/CT data [12]

	Standard of care	PSMA-PET/CT strategy	Difference
PI-RADS 1–2			
* Quality adjusted life years*	12.976	12.975	−0.001
* Metastases, n*	81	75	−6
* Diagnostic costs, €*	€ 985,00	€ 1.381,00	€ 396,00
* Treatment costs, €*	€ 2.889,00	€ 3.263,00	€ 374,00
* Long term costs, €*	€ 18.766,00	€ 18.425,00	-€ 341,00
* Total disease costs, €*	€ 22.640,00	€ 23.069,00	€ 429,00
* ICER*	0	Not applicable	
PI-RADS 3			
* Quality adjusted life years*	12.976	12.978	+ 0.002
* Metastases, n*	81	83	2
* Diagnostic costs, €*	€ 985,00	€ 1.182,00	€ 197,00
* Treatment costs, €*	€ 2.889,00	€ 2.816,00	-€ 73,00
* Long term costs, €*	€ 18.766,00	€ 18.828,00	€ 62,00
* Total disease costs, €*	€ 22.640,00	€ 22.826,00	€ 186,00
* ICER*	0	€ 93.212,00	Borderline cost-effective
PI-RADS 1–3			
* Quality adjusted life years*	12.976	12.977	+ 0.001
* Metastases, n*	81	76	−5
* Diagnostic costs, €*	€ 985,00	€ 1.578,00	€ 593,00
* Treatment costs, €*	€ 2.889,00	€ 3.190,00	€ 301,00
* Long term costs, €*	€ 18.766,00	€ 18.487,00	-€ 279,00
* Total disease costs, €*	€ 22.640,00	€ 23.255,00	€ 615,00
* ICER*	0	€ 615.287,00	Not cost-effective

*CT*, computed Tomography; *ICER*, Incremental cost-effectiveness ratio; *PET*, Positron Emission Tomography; *PI-RADS*, Prostate Imaging Reporting and Data System; *PSMA*, Prostate-Specific Membrane Antigen

**Table 9:** Quality of life benefit and cost-effectiveness using external MRI [13] and external PSMA-PET/CT data [11]

	Standard of care	PSMA-PET/CT strategy	Difference
PI-RADS 1–2			
* Quality adjusted life years*	12.976	12.976	+ 0.001
* Metastases, n*	81	76	−5
* Diagnostic costs, €*	€ 985,00	€ 1.397,00	€ 412,00
* Treatment costs, €*	€ 2.889,00	€ 3.209,00	€ 320,00
* Long term costs, €*	€ 18.766,00	€ 18.459,00	-€ 307,00
* Total disease costs, €*	€ 22.640,00	€ 23.065,00	€ 425,00
* ICER*	0	€ 424.814,00	Not cost-effective
PI-RADS 3			
* Quality adjusted life years*	12.976	12.980	+ 0.004
* Metastases, n*	81	84	3
* Diagnostic costs, €*	€ 985,00	€ 1.197,00	€ 212,00
* Treatment costs, €*	€ 2.889,00	€ 2.772,00	-€ 117,00
* Long term costs, €*	€ 18.766,00	€ 18.854,00	€ 88,00
* Total disease costs, €*	€ 22.640,00	€ 22.823,00	€ 183,00
* ICER*	0	€ 45.686,00	Cost-effective
PI-RADS 1–3			
* Quality adjusted life years*	12.976	12.980	+ 0.004
* Metastases, n*	81	78	−3
* Diagnostic costs, €*	€ 985,00	€ 1.608,00	€ 623,00
* Treatment costs, €*	€ 2.889,00	€ 3.093,00	€ 204,00
* Long term costs, €*	€ 18.766,00	€ 18.547,00	-€ 219,00
* Total disease costs, €*	€ 22.640,00	€ 23.247,00	€ 607,00
* ICER*	0	€ 151.890,00	Not cost-effective

*CT*, computed Tomography; *ICER*, Incremental cost-effectiveness ratio; *PET*, Positron Emission Tomography; *PI-RADS*, Prostate Imaging Reporting and Data System; *PSMA*, Prostate-Specific Membrane Antigen

## Discussion

It is hypothesized that the use of PSMA-PET/CT in addition to prostate MRI could improve the detection of prostate cancer as it may unveil additional tumors in negative MRI examinations (PI-RADS 1–2) and could avoid biopsies in men with equivocal MRI findings (PI-RADS 3). Yet, it was unknown whether this strategy would be cost-effective and improve patients' quality-of-life. We showed here that additional PSMA-PET/CT can improve the quality of life and may be cost-effective in PI-RADS 3 patients. However, in PI-RADS 1–2 patients, it does not enhance the quality of life nor is it cost-effective.

PI-RADS 3 lesions are frequently seen with prostate MRI. Particularly if no highly qualified and dedicated MRI team is available, the PI-RADS 3 rate more than triples from 6 to 21% [15, 16]. This leads to an excessive number of unnecessary biopsies. We observed that the MRI + PSMA-PET/CT pathways would avoid biopsies in 21 or 24% of patients (Table 6), and thus a majority of PI-RADS 3 classifications/assessments. By preventing biopsies, the MRI + PSMA-PET/CT pathways could potentially improve the overall quality of life. Moreover, in the last decade, there has been a shift towards not treating low-risk prostate cancers. Therefore, it is presently preferred that these clinically insignificant cancers remain undetected as it reduces cancer anxiety and over-treatment (10). In the setting of a PI-RADS 3 lesion, the MRI + PSMA-PET/CT pathway could have prevented detection of 5 or 7% of these low-risk cancers and by doing so, could have improved the quality of life. When an additional PSMA-PET/CT was performed in negative MRIs (PI-RADS 1–2), the opposite effect was observed. The number of unnecessary biopsies would have increased (+ 11 or 15%) with only a minor increase of the detection of clinically significant prostate cancers (0 or 4% high risk and 10 or 13% of intermediate risk prostate cancers). These could potentially also have been detected during follow-up with PSA testing.

To date, PET/CT is expensive requiring costly machinery, a specialized workforce and management for radiation safety. While there is incremental access for this modality in developed countries and as a result, a reduction in costs [17]. Its availability is significantly lower and more expensive compared to MRI or biopsies. In our model, the additional costs per patient of PSMA-PET/CT following PI-RADS 1–2 or PI-RADS 3 were €416—€429 and €170—€186, respectively. Due to the improvement in QALY with the reduction in biopsies and less detection of low risk prostate cancers, the strategy is borderline cost-effective in PI-RADS 3 with an ICER of €56,700 – €93,212. However, it is more expensive than the standard of care. For the new strategy in PI-RADS 3 to be cost-beneficial, the price needs to be similar to the price of the MRI and biopsy. This would require a ~ 50% reduction in price for PSMA PET/CT.

In case of a negative MRI (PI-RADS 1–2), the MRI + PSMA-PET/CT pathway was not cost-effective. As the sensitivity for MRI is high in experienced centers, we do not believe there is a benefit performing PSMA PET/CT in unselected PI-RADS 1–2 patients. However, in selected patients with persistent suspicion of prostate cancer and (repeatedly) negative MRI results, PSMA-PET/CT may be beneficial by identifying lesions for targeted biopsy instead of random sampling. However, this hypothesis needs to be validated in a larger trial.

To validate our findings, we conducted an additional analysis using data from an external PSMA-PET/CT study in biopsy naïve men (PRIMARY1 study), which confirmed our results and strengthened the conclusions about the clinical and economic implications of the diagnostic strategy. The PSMA-PET/CT was found to be cost-effective in PI-RADS 3 cases but not in PI-RADS 1–2, despite slightly improving quality of life by potentially preventing metastases. However, this benefit was offset by numerous unnecessary biopsies and over-detection of low-risk prostate cancers. We thus believe, looking at the present models, the PI-RADS 3 cohort has the largest benefit of PSMA-PET/CT in this setting. In the PRIMARY2 study (NCT05154162), which is a currently recruiting non-inferiority trial (powered on the PRIMARY1 study outcomes) of the prostate MRI plus PSMA-PET/CT to detect clinically significant prostate cancer, only PI-RADS 3 and PI-RADS 2 patients with high-risk features are allowed to participate (PSA density > 0.1 ng/ml^2^, abnormal digital rectal exam, strong family history, BRCA mutation, PSA > 10, PSA doubling time < 36 months, PSA velocity > 0.75 ng/ml/yr). With these rather broad inclusion criteria for PI-RADS 2, one may still question whether this strategy will be cost-effective.

Our study has several limitation. First, despite utilizing different input models, the PSMA-PET/CT outcomes are based on a limited patient sample. Therefore, the exact numbers may change using a larger cohort and the phase 3 data is highly anticipated to support the present findings. Second, our initial study and the external PRIMARY1 trial used different tracers ([^18^F]PSMA-1007 vs. [^68^Ga]Ga-PSMA-11), which could lead to different scan results. Third, the current model used Dutch healthcare costs and since intervention costs vary by country, this can impact cost-effectiveness outcomes. Moreover, at €1129 per PSMA-PET/CT, we are at the lower price range. To expand PSMA-PET/CT use in earlier settings, strategies to further reduce costs, such as using one bed position scans and lower injected activities, are needed.

## Conclusion

The addition of PSMA-PET/CT to MRI in patients with equivocal MRI findings appears to be borderline cost-effective due to biopsy avoidance and a reduced detection of indolent, low-risk tumors. In men with a negative MRI, adding a PSMA-PET/CT does not seem to be cost-effective due to a higher number of unnecessary biopsies and only minor improvement in the detection of clinically significant prostate cancer.

## Supplementary Information

Below is the link to the electronic supplementary material.

Supplementary file1 (DOCX 738 KB)

## Data Availability

The data that support the findings of this study are available from the corresponding author upon reasonable request.
