# Supplementary material for: A cost-effectiveness study of PSMA-PET/CT for the detection of clinically significant prostate cancer
Source: Eur J Nucl Med Mol Imaging. 2025 Mar 12;52(9):3159–69. doi: 10.1007/s00259-025-07190-6 (PMC12222398; doi:10.1007/s00259-025-07190-6)
Supplement: Supplementary file 1 — Supplementary file1 (DOCX 738 KB) [file 259_2025_7190_MOESM1_ESM.docx]

**Supplementary figure 1: Tornado plot with the effect of a 25% decrease/increase in model inputs**
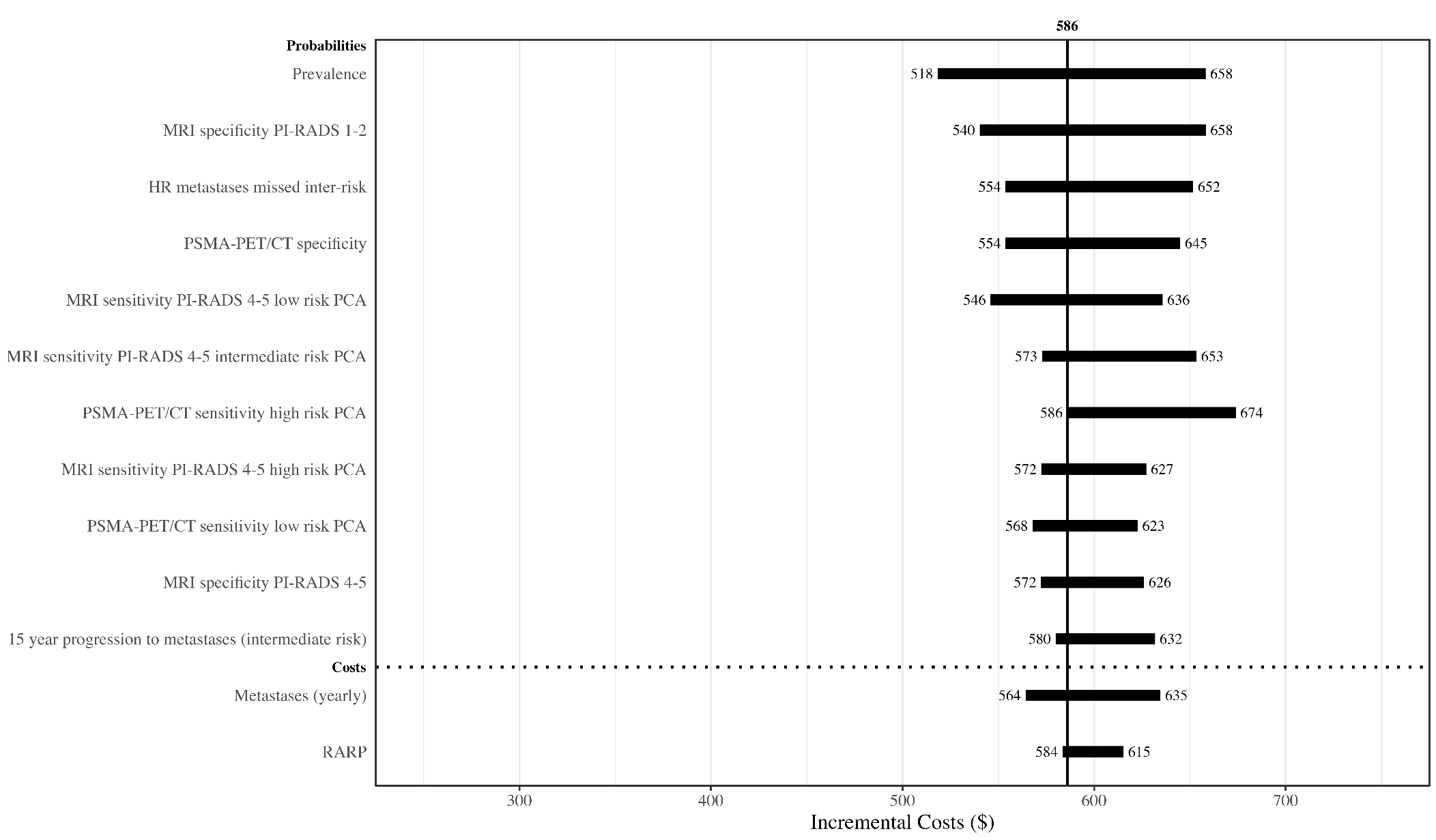


Data was based on the MRI data of the PICTURE trial in PI-RADS 1-3. Everything with less than 5% impact on the outcome were excluded from the tornado plot. CT = computed tomography MRI = magnetic resonance imaging; PCA = prostate cancer; PI-RADS = Prostate Imaging–Reporting and Data System; PSMA = prostate specific membrane antigen; PET = positron emission tomography;

**Supplementary figure 2: Tornado plot with the effect of a 25% decrease/increase in model inputs**
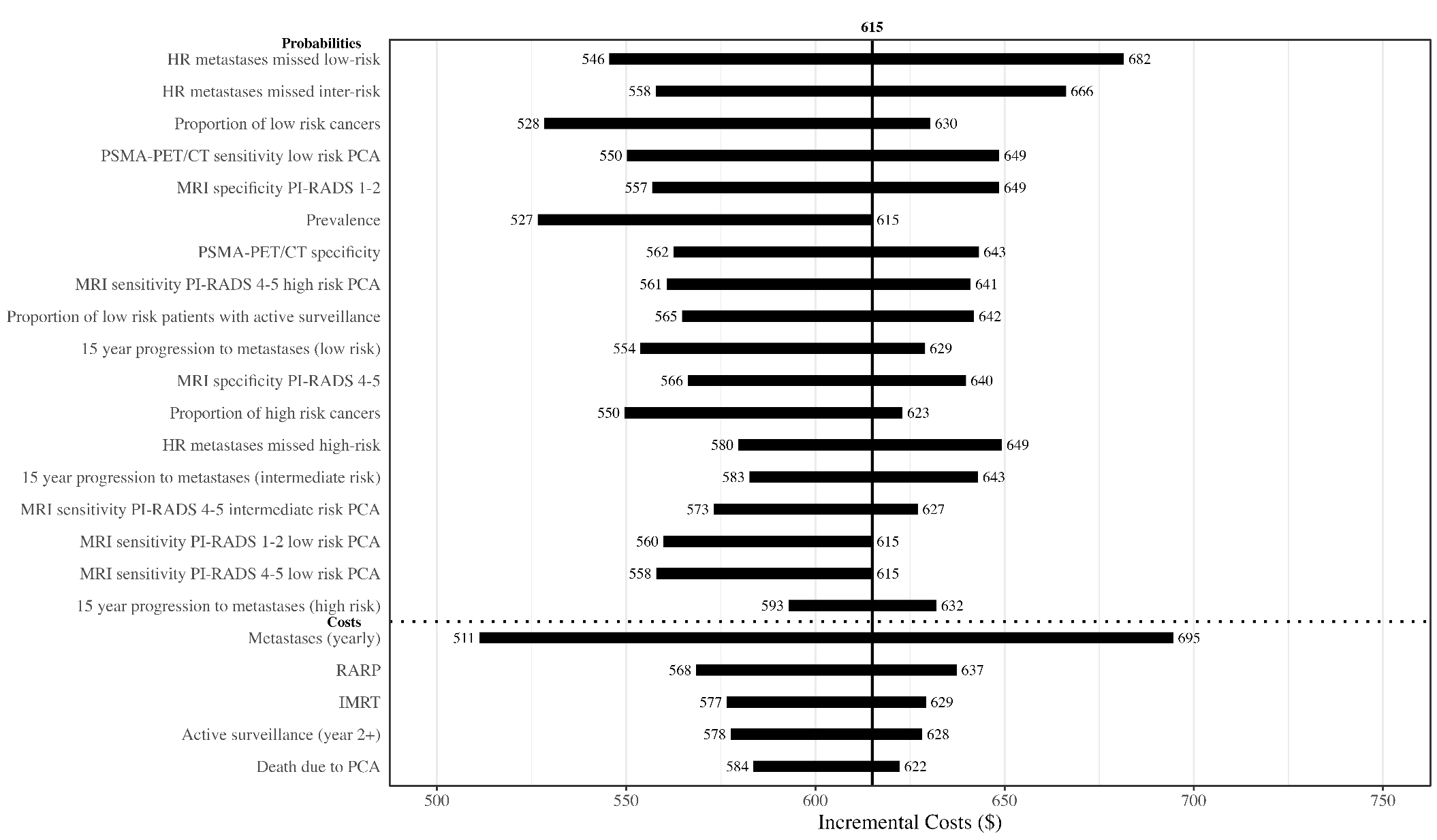


Data was based on the MRI data of the Drost et al, 2020 in PI-RADS 1-3. Everything with less than 5% impact on the outcome were excluded from the tornado plot. CT = computed tomography; IMRT = Intensity Modulated Radiotherapy; MRI = magnetic resonance imaging; PCA = prostate cancer; PI-RADS = Prostate Imaging–Reporting and Data System; PSMA = prostate specific membrane antigen; PET = positron emission tomography; RARP = Robot Assisted Radical Prostatectomy

|  | Initial study participants  (n= 75) | External validation study participants (n= 291) |
| --- | --- | --- |
| Age, years, median (IQR) | 67 (62-70) | 64.0 (59 - 80) |
| Suspicious DRE, yes, n (%) | 24 (32) | 86 (26.5) |
| PSA at inclusion (ng/ml), median (IQR) | 7 (4.9-10) | 5.6 (4.2 - 7.5) |
| Volume of prostate (mpMRI), median (IQR) | 51 (41-74) | 40 (29 – 55) |
| No cancer | 57 (56) * | 77 (26) # |
| ISUP 1 | 20 (20) * | 52 (18) # |
| ISUP 2 | 20 (20) * | 102 (35) # |
| ISUP 3 | 3 (3) * | 39 (13) # |
| ISUP 4 | 1 (1) * | 7 (2.4) # |
| ISUP 5 | 1 (1) * | 14 (4.8) # |

DRE = digital rectal exam; ISUP ISUP = International Society of Urological Pathology; * Lesion based, # patient based

**References**

Privé BM, Israël B, Janssen MJR, et al. Multiparametric MRI and ^18^F-PSMA-1007 PET/CT for the Detection of Clinically Significant Prostate Cancer. *Radiology*. 2024;311(2):e231879. doi:10.1148/radiol.231879

Emmett L, Buteau J, Papa N, et al. The Additive Diagnostic Value of Prostate-specific Membrane Antigen Positron Emission Tomography Computed Tomography to Multiparametric Magnetic Resonance Imaging Triage in the Diagnosis of Prostate Cancer (PRIMARY): A Prospective Multicentre Study. Eur Urol. 2021;80(6):682-689. doi:10.1016/j.eururo.2021.08.002
